# Acute thrombocytopenia induced by trastuzumab due to complement reaction: A case report

**DOI:** 10.3389/fmed.2022.1037493

**Published:** 2022-12-06

**Authors:** Guoping Chen, Jianghua Ou, Jun Liu, Haoran Liao, Linwei Ding, Pingming Fan, Guankui Du

**Affiliations:** ^1^Department of Breast Surgery, Affiliated Tumor Hospital of Xinjiang Medical University, Ürümqi, China; ^2^Department of Breast Surgery, The First Affiliated Hospital of Hainan Medical University, Haikou, China; ^3^Department of Immunology, Hainan Medical University, Haikou, China; ^4^Key Laboratory of Molecular Biology, Hainan Medical University, Haikou, China; ^5^Department of Biochemistry and Molecular Biology, Hainan Medical University, Haikou, China; ^6^Biotechnology and Biochemistry Laboratory, Hainan Medical University, Haikou, China

**Keywords:** breast cancer, HER2, trastuzumab, dramatic thrombocytopenia, complement response

## Abstract

**Background:**

The usual treatment option for HER2 breast cancer is targeted therapy with trastuzumab. The common adverse effects of trastuzumab treatment are thrombocytopenia, however, acute thrombocytopenia is rare and its mechanism is still largely unknown.

**Case presentation:**

We reported a patient who presented with acute thrombocytopenia on two consecutive occasions, and the predisposing factor was identified on the second occasion because of trastuzumab-only treatment. Routine blood results showed a dramatic increase in white blood cell count and neutrophil count after both trastuzumab treatments. Moreover, the complement reaction results suggested that the dramatic thrombocytopenia was probably due to platelet destruction after complement activation.

**Conclusion:**

This case suggests that it would be useful to perform a platelet complement reaction test before trastuzumab treatment in patients with HER2 breast cancer.

## Introduction

Breast cancer in women has surpassed lung cancer as the most common cancer, with an estimated 2.3 million new cases (11.7%) (1). Breast cancer is a heterogeneous disease with multiple subtypes, each of which (Luminal type, HER2 + type, triple-negative breast cancer) all have unique clinical, pathological, and molecular features (2, 3). HER2 is an epidermal growth factor receptor that is highly expressed in 20% of breast cancers (4). Anti-HER2 therapy has become the standard treatment for breast cancer patients with high HER2 expression, and a variety of anti-HER2 drugs, including trastuzumab, have achieved good clinical results (5).

Trastuzumab is 95% humanized and has low allergenicity (5). Common adverse reactions are infusion-related symptoms, including chills, fever, pain, vomiting, fatigue, etc., which mostly occur after the first medication (6). Thrombocytopenia is also induced by trastuzumab, a common grade 3 or higher adverse event (7).

Severe thrombocytopenia due to trastuzumab is rare, and only a small number of cases have been reported (8–10). The cause of severe thrombocytopenia with trastuzumab is unknown. Here, we report a case from our hospital and analyze the possible mechanism of trastuzumab-induced severe thrombocytopenia.

## Case description

A 51-year-old woman, 55 kg, 155 cm, presented with a left breast tumor. The local skin has no redness, swelling, ulceration, or “orange peel-like” change, without pain, nipple bleeding, and nipple depression. The examination revealed a mass of about 3.0 cm × 2.0 cm, which was hard in texture, with poorly defined borders, a less smooth surface, and no tenderness. An enlarged lymph node of about 1.5 cm × 0.5 cm was found in the left axilla. The immunohistochemical pathology report suggested invasive unspecified carcinoma. E-cadherin (membrane +), ER (weak +, 5%), PR (weak +, 10%), CK5/6 (-), Her-2 (3 +), P120 (membrane +), Ki (+, 40%).

Medical history: No previous history of the disease. No family history of genetic predisposition. No preoperative history of blood transfusion. No psychosocial history.

Six cycles of neoadjuvant chemotherapy (docetaxel 120 mg, epirubicin 120 mg, and cyclophosphamide 950 mg) were performed in the TEC regimen. During chemotherapy, neutropenia occurred after IV chemotherapy, and it improved after treatment with recombinant human granulocyte-stimulating factor. The results of physical examination showed that the two breasts were symmetrical after chemotherapy, there was no obvious mass in both breasts, and there was no enlargement in axillary and supraclavicular lymph nodes. Dynamic re-examination of breast color Doppler ultrasound indicated that the mass was reduced. No platelet drop was observed during chemotherapy. Three months later, a modified radical left breast cancer was performed and intraoperative Lobaplatin 60 mg was administered to irrigate the wound. Five days after the modified radical mastectomy for breast cancer, the incision was well healed and the drainage tube was not removed from the operated area. The efficacy was evaluated as PR.

**FIGURE 1:**
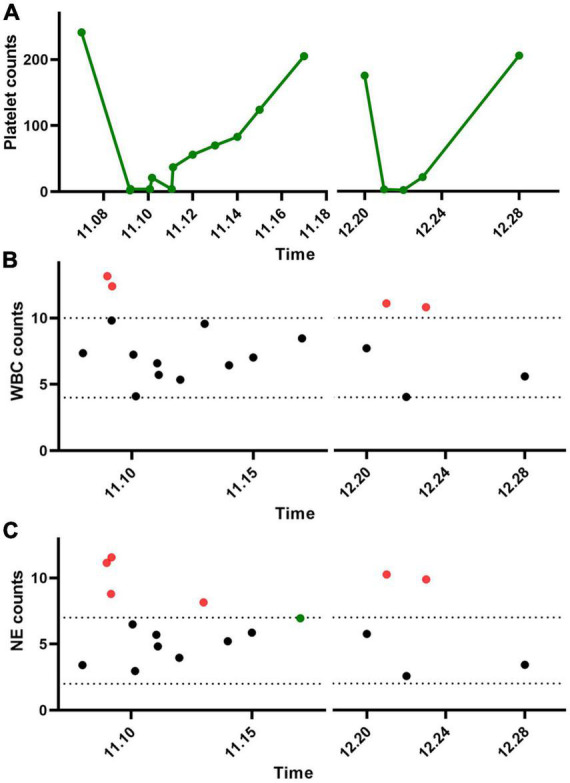
Changes in platelets and leukocytes after two trastuzumab treatments in the patient. **(A)** Platelets, **(B)** white blood cells, and **(C)** neutrophils.

Trastuzumab targeted therapy was given for the first time 3 days after surgery. Pre-treatment routine blood tests showed normal. A total of 8 mg/kg trastuzumab was administered intravenously. Six hours after infusion, there was gingival bleeding, and subcutaneous ecchymosis in the operation area, and the red liquid was drawn out from the drainage tube. Routine blood tests showed a WBC count of 9.82 × 10^9/L, a PLT count of 2 × 10^9/L, and an NE count of 8.79 × 10^9/L (Figures 1A–C). Four coagulation tests showed normal. Meanwhile, a bone marrow puncture was performed to exclude the primary decrease in platelet. Fresh frozen plasma and platelets of the same type were transfused, and human immunoglobulin injection and recombinant human thrombopoietin were added. The patient gradually returned to normal.

One month later, the patient returned to the hospital for targeted therapy. Before treatment, routine blood tests were normal, platelet count was normal, and the four coagulation tests were normal. No other medication was given. A total of 6 mg/kg trastuzumab was administered intravenously. Gum bleeding with scattered petechiae and petechiae under the skin reappeared at 6 h. The routine blood test showed that the WBC count was 11.10 × 10^9/L, HGB count was 122 g/L, PLT count was 3 × 10^9/L, and NE count was 10.26 × 10^9/L (Figures 1A–C). The four coagulation tests showed normal. He improved after symptomatic treatment with human immunoglobulin, blood transfusion, dexamethasone, and recombinant human platelet thrombopoietin.

**FIGURE 2:**
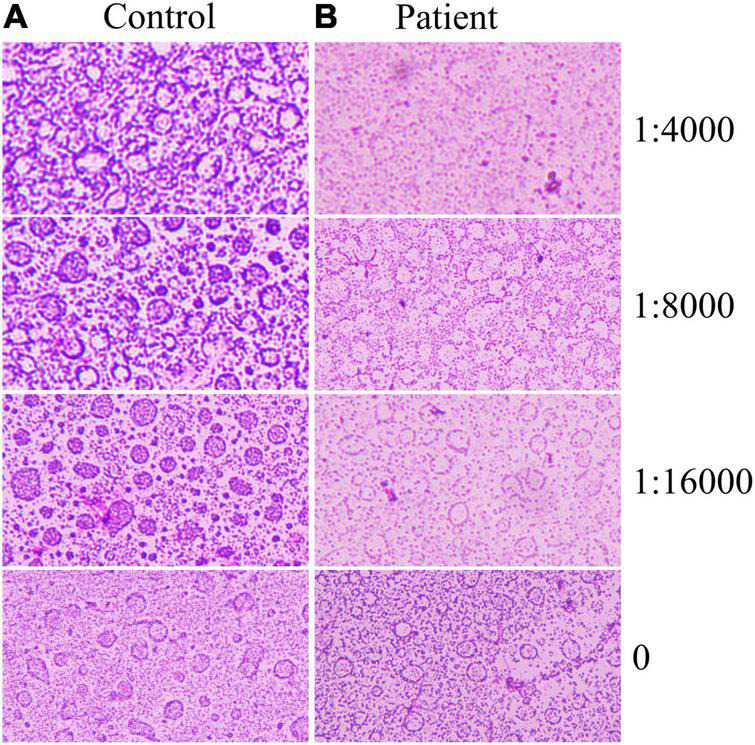
Platelet complement reaction. Trastuzumab was diluted 16,000, 8,000, and 4,000 times, and different samples of platelets reacted with biological complement. **(A)** Trastuzumab-treated controls without acute hemolysis. **(B)** Trastuzumab in patients with acute hemolysis.

Since sharp increases in leukocytes and neutrophils in patients, it is hypothesized that the complement reaction is the main cause of the dramatic decrease in platelets. Therefore, the extracted platelets were subjected to a complement hemolysis assay (Figure 2). It was shown that a large number of platelets from the patient were destroyed after the addition of trastuzumab and complement. In contrast, platelets in other HER2 breast cancer patients maintained normal morphology.

The patient was rechecked every 3 months after discharge, and the blood routine, liver and kidney function, and tumor markers were normal. No further decline in platelets was detected. The patient’s compliance was good During the follow-up with no adverse or unexpected conditions.

## Discussion

Multiple causes can lead to thrombocytopenia, such as bacterial and viral infections, liver disease, kidney disease, alcohol abuse, pregnancy, and the use of medications (11–15). Pharmacogenetic thrombocytopenia is a sharp drop in platelet count that occurs in patients during the use of drugs. Epidemiological studies have shown that the annual incidence of pharmacogenetic thrombocytopenia is 10 cases/1 million individuals (16). Patients with severe thrombocytopenia are at increased risk of microtraumatic bleeding and spontaneous bleeding, which endanger health. Drug-induced reduction in platelet count is mainly through two pathways: inhibition of platelet production, including inhibition of differentiation and maturation of megakaryocytes and mature shedding of platelets; induction of specific antibody production, which destroys platelets through an immune response (17). The case report demonstrated a patient with HER2 high expression breast cancer who developed symptoms of acute thrombocytopenic purpura after the use of trastuzumab.

Trastuzumab is a monoclonal antibody that blocks the growth of cancer cells by attaching to HER2 and preventing the binding of human epidermal growth factor to HER2 (18). Trastuzumab is mainly used clinically for metastatic breast cancer with overexpression of HER2 (18). Trastuzumab has been shown to inhibit the proliferation of HER2-overexpressing tumor cells both *in vitro* and in animal studies (19, 20). Additionally, trastuzumab is a potential mediator of antibody-dependent cell-mediated cytotoxicity (21, 22). In the present case report, trastuzumab was the drug of choice because the patient’s pathological findings showed a high expression of HER2.

Studies show that trastuzumab adverse reactions are mostly mild thrombocytopenia (23, 24), and the incidence of thrombocytopenia in treated patients is about 25 to 31%, with an incidence of grade ≥3 of about 2–15% (25). The risk of thrombocytopenia is higher in the Asian population, up to 52.5–69.8%, and the incidence of grade ≥3 is about 29.8–45% (25). Studies have shown that trastuzumab generally has the lowest platelet count on day 8 of dosing (26). At present, a few cases of acute thrombocytopenia induced by trastuzumab have been reported. The youngest patient was 29 years old and half of the patients were over 50 years old (27). Seven patients developed acute thrombocytopenia on the first dose of trastuzumab, most of which occurred within 24 h (9). A patient developed acute thrombocytopenia about 10 h after the first dose of trastuzumab (9). The rest of the patients developed symptoms at 1–10 days (10, 28, 29). Trastuzumab-induced acute thrombocytopenia resulted in petechiae, nosebleeds, dental bleeding, and uterine bleeding (10). The present case report demonstrated a patient with HER2-positive breast cancer who developed acute thrombocytopenia at about 6 h on the first dose of trastuzumab. Multiple drugs were administered concurrently with this treatment and it is not certain that the symptoms were induced by trastuzumab. The second treatment, with only trastuzumab, reappeared with acute thrombocytopenia at about 6 h, thus confirming that the symptoms were due to trastuzumab treatment. In this case, the patient had two consecutive episodes of thrombocytopenic purpura, the first of which could not be determined to be due to trastuzumab induction because of interference from other drugs. The second time only trastuzumab was used. Therefore, this case report clarifies that trastuzumab can induce thrombocytopenic purpura in some patients.

The mechanism of trastuzumab-associated thrombocytopenia has not been clarified. Studies have shown that it may be related to the endocytosis of trastuzumab in megakaryocytes (30). The intracellular release of trastuzumab after endocytosis affects the differentiation of megakaryocytes, ultimately leading to the impairment of megakaryocyte maturation and platelet generation (27). Neutrophils are important members of white blood cells and they can rapidly participate in early innate immune responses due to their accumulation in bone marrow reservoirs (31). After vascular injury and endothelial activation, P-selectin on the platelet surface interacts with P-selectin protein-ligand 1 expressed by neutrophils to activate neutrophils and improve their phagocytosis, lethality, and clearance rate (32, 33). Neutrophils interacting with platelets can phagocytose platelets and also promote the generation of neutrophil extracellular traps (NETs) (34). NETs provide a scaffold for thrombosis and promote thrombosis and coagulation (35). In the present case, the patient’s white blood cells and neutrophil levels increased dramatically during the initial phase of trastuzumab-induced thrombocytopenia. After the remission of symptoms, white blood cell and neutrophil levels returned to normal. Therefore, it is speculated that the dramatic increase in neutrophils may be associated with a sharp decrease in platelets, the mechanism of which deserves further investigation.

The complement system is an important part of innate immunity and consists of more than 40 activating proteins, regulators, and receptors on vascular cells (36). Activation of the complement system mediates the immune response and inflammatory reaction (37). Antigen-antibody complexes can activate complement, and the activated complement components can combine with antigen-antibody complexes, resulting in a series of immunological reactions, such as cytotoxic/bacteriolytic reactions and hemolytic reactions (36). In this case report, a complement reaction assay was performed with the patient’s consent. The results showed that the platelets of control patients (HER +, Treated with trastuzumab) did not decrease after the addition of trastuzumab. On the contrary, the platelets of the patient in this case decreased dramatically after the addition of low concentrations of trastuzumab. Since trastuzumab is a HER2-specific antibody, this result indirectly reflects that the patient’s platelets are enriched in HER2. Therefore, this case highlights that the acute thrombocytopenia may be due to the trastuzumab-HER2-mediated complement reaction.

## Conclusion

This case report is the first to elucidate the risk of trastuzumab, a specific antibody to HER2 protein, to induce a complement response leading to acute thrombocytopenia. Therefore, the results of the case indicate that a simple platelet complement reaction is an option for analyzing the applicability of trastuzumab before HER2-positive breast cancer patients receive treatment.

## Data availability statement

The raw data supporting the conclusions of this article will be made available by the authors, without undue reservation.

## Ethics statement

The studies involving human participants were reviewed and approved by the Ethics Committee of the First Affiliated Hospital of Hainan Medical College (2022kyl153). The patients/participants provided their written informed consent to participate in this study.

## Author contributions

GC contributed to the funding acquisition and investigation. GD contributed to the methodology, project administration, resources, and writing – original draft and editing. HL and PF contributed to the software. JL and LD contributed to the conceptualization and formal analysis. PF contributed to the writing – original drafts and editing. All authors contributed to the article and approved the submitted version.
